# Immunogenicity of a universal HIV-1 vaccine vectored by DNA, MVA and CHADV-63 in a Phase I/IIA clinical trial

**DOI:** 10.1186/1742-4690-9-S2-P118

**Published:** 2012-09-13

**Authors:** NJ Borthwick, T Ahmed, A Rose, U Ebrahimsa, A Black, E Hayton, H Yang, G Hancock, S Campion, N Frahm, S Colloca, A Nicosia, A McMichael, L Dorrell, T Hanke

**Affiliations:** 1 University of Oxford , Oxford, UK; 2 University of Oxford, Human Immunology Unit, Oxford, UK; 3 HIV Vaccine Trials Network, University of Washington, Seattle, USA; 4 Okairos, Rome, Italy; 5 Oxford University, Human Immunology Unit, Oxford, UK

## Background

The major challenge facing both antibody and T cell-eliciting vaccines against HIV-1 is the extreme variability of the HIV-1 genome: a successful vaccine has to effectively target diverse HIV-1 strains circulating in the population and then must deal with ongoing virus escape in infected individuals. To address these issues, we assembled a vaccine immunogen HIVconsv from the functionally most conserved regions (not epitopes) of the HIV-1 proteome.

## Methods

A gene coding for the HIVconsv immunogen was inserted into plasmid DNA (D), modified vaccinia virus Ankara (MVA; M) and non-replicating adenovirus of a chimpanzee origin ChAdV-63 (C). Currently, combined heterologous prime-boost regimens of these vaccines, namely CM, DDDCM and DDDMC, are being evaluated in a phase I/IIa trial HIV-CORE002 in healthy HIV-1/2-negative volunteers in Oxford.

## Results

Preliminary data indicate that the vaccines are well tolerated and show high immunogenicity. Following the CM regimen, vaccine-induced T cell frequencies reached a median of 5150 (range 1475 to 16495) SFU/10^6^ PMBC ex vivo one week post MVA vaccination. DNA priming increased subsequent T cell responses to ChAdV-63 vaccination (median: C 577 and DDDC 1328 SFU/10^6^ PBMC) and ELISpot responses again peaked 1 week following MVA (median 4500; range 2260-7960 SFU/10^6^ PBMC). Matrix analyses of the participants following CM vaccination showed that T cells responded to a range of peptides across the length of HIVconsv. The CM regimen elicited IFN-γ in both CD4+ and CD8+ T cell subsets and polyfunctional (IFN-γ & TNF-α) responses to HIVconsv peptides.

## Conclusion

Presented data will be very much work in progress. Nevetheless, the HIVconsv vaccines have so far induced T cell responses superior to other HIV-1 vaccine candidates tested to date. ChAdV-63 is the first adenovirus of chimp origin delivering an HIV-1-derived immunogen that has reached the clinic.

The work is supported by Medical Research Council UK.

